# Metabolite Diversity and Metabolic Genome-Wide Marker Association Studies (Mgwas) for Health Benefiting Nutritional Traits in Pearl Millet Grains

**DOI:** 10.3390/cells10113076

**Published:** 2021-11-08

**Authors:** Chandra Bhan Yadav, Rakesh K. Srivastava, Prakash I. Gangashetty, Rama Yadav, Luis A. J. Mur, Rattan S. Yadav

**Affiliations:** 1 Institute of Biological Environmental and Rural Sciences (IBERS), Aberystwyth University, Plas Gogerddan, Aberystwyth SY23 3 EB, UK; chy10@aber.ac.uk (C.B.Y.); ray7@aber.ac.uk (R.Y.); lum@aber.ac.uk (L.A.J.M.); 2 International Crops Research Institute for the Semi-Arid Tropics, Patancheru, Hyderabad 502324, India; r.k.srivastava@cgiar.org (R.K.S.); P.Gangashetty@cgiar.org (P.I.G.)

**Keywords:** pearl millet, metabolites, starch, antioxidants, vitamins, germplasm, mGWAS, marker-trait associations, candidate genes

## Abstract

As efforts are made to increase food security, millets are gaining increasing importance due to their excellent nutritional credentials. Among the millets, pearl millet is the predominant species possessing several health benefiting nutritional traits in its grain that are helpful in mitigating chronic illnesses such as type−2 diabetes and obesity. In this paper, we conducted metabolomic fingerprinting of 197 pearl millet inbred lines drawn randomly from within the world collection of pearl millet germplasm and report the extent of genetic variation for health benefitting metabolites in these genotypes. Metabolites were extracted from seeds and assessed using flow infusion high-resolution mass spectrometry (FIE-HRMS). Metabolite features (*m*/*z*), whose levels significantly differed among the germplasm inbred lines, were identified by ANOVA corrected for FDR and subjected to functional pathway analysis. A number of health-benefiting metabolites linked to dietary starch, antioxidants, vitamins, and lipid metabolism-related compounds were identified. Metabolic genome-wide association analysis (mGWAS) performed using the 396 *m*/*z* as phenotypic traits and the 76 K SNP as genotypic variants identified a total of 897 SNPs associated with health benefiting nutritional metabolite at the -log *p*-value ≤ 4.0. From these associations, 738 probable candidate genes were predicted to have an important role in starch, antioxidants, vitamins, and lipid metabolism. The mGWAS analysis focused on genes involved in starch branching (α-amylase, β-amylase), vitamin-K reductase, UDP-glucuronosyl, and UDP-glucosyl transferase (UGTs), L-ascorbate oxidase, and isoflavone 2′-monooxygenase genes, which are known to be linked to increases in human health benefiting metabolites. We demonstrate how metabolomic, genomic, and statistical approaches can be utilized to pinpoint genetic variations and their functions linked to key nutritional properties in pearl millet, which in turn can be bred into millets and other cereals crops using plant breeding methods.

## 1. Introduction

Globally, more than half of all deaths are due to malnutrition, and this is driving the need to improve the nutritional content of staple food crops that are consumed on a daily basis. Of the many edible plants available, cereals and millets contribute more than 50 % of human energy intake. Millets are naturally rich in both micronutrients as well as several other health-benefiting characteristics. Nutrition-rich products derived from millets, therefore, have the potential to counter human malnutrition. Nowadays, millets are grown worldwide, but were domesticated mainly in China, Asia, and Africa. Millets are C4 plant species and so have higher photosynthetic rates compared to other C3 plants [1,2]. They also display better water use efficiency than other members of grass families such as maize, wheat, and sorghum [3]. Millets are usually referred to as coarse grains; however, due to their important nutritional properties, they are now being termed as ‘nutria-millets/nutria-cereals’ as well. Most of the millets, including pearl millet, have high protein, folic acid, vitamins, carotenoid contents [4,5], possess non-glutinous and non-acid forming properties, and are easy to digest. They are also rich in nutritionally important minerals like iron, calcium, zinc, magnesium, phosphorous, and potassium [6,7,8], and dietary fiber and several vitamins (β-Carotene, niacin, vitamin B6 and folic acid) [9,10]. Millets are also rich in polyphenols, tannins, phytosterols and are a good source of antioxidants. Despite these positive attributes, the use of millets as a food is restricted to the conventional consumers, predominantly the subsistence farming families who grow them. This can be attributed to the non-availability of user-friendly, ready to use/ready to eat millet-based food products.

The value of millets is specifically underlined by their health benefits. Several research studies have shown that diets that are dominated by millet grains can contribute to the protection against several non-communicable diseases (NCDs) such as cancer, cardiovascular ailments, diabetes, metabolic syndrome, and Parkinson’s disease [11,12,13]. Until recently the vitamins, minerals, essential fatty acids, and fiber in grains were considered as the important constituents, however, new research suggests that the combination of other bioactive substances they possess are also important for health benefits [14]. These include slowly digestible and resistant starches; oligosaccharides; lipids; antioxidants, and flavonoids; and antinutrients such as phytic acid and tannins [15,16].

The development of pearl millets varieties to maximize their value as a functional food and nutraceuticals is being complemented by the availability of the pearl millet (*Pennisetum glaucum*) genome sequence [4]. Recent genomic studies have successfully provided the genetic architecture of complex traits like drought tolerance, N_2_ use efficiency, C4 photosynthetic efficiency, and bioenergy properties [5]. The availability of the genomic sequence is also allowing millets to be used as model plants as they have relatively a short life cycle. The power of pearl millet genomic studies can be augmented if coupled with metabolomics, which is emerging as an important analytical approach in food and nutrition research [17]. Currently, a number of metabolomics platforms such as liquid chromatography-mass spectrometry (LC-MS), gas chromatography-mass spectrometry (GC–MS), nuclear magnetic resonance (NMR), flow infusion electrospray ionization high-resolution mass spectrometry (FIE-HRMS) are accelerating metabolite fingerprinting in a stated sample [18]. Of these, FIE-HRMS is one of the most accurate, reliable analytical methods to detect volatile and thermally stable compounds, including sugars, amino acids, organic acids, and polyamines, as well as biosynthetic pathway precursors [19,20]. Dissection of the genetic bases for food and nutritional metabolites has also allowed the global identification of genetic determinants for the diversity of plant metabolism.

The present study assessed metabolic diversity in nutraceutical elements using a subset of randomly picked 197 genotypes drawn from within the world collection of 345 Pearl Millet inbred Germplasm Association Panel (PMiGAP) [21]. Using a metabolic genome-wide association study (mGWAS) approach, we report candidate genes associated with a diversity of plant metabolites in this germplasm set.

## 2. Materials and Methods

### 2.1. Plant Materials

A total of 197 genotypes were picked randomly from the pearl millet inbred germplasm association panel (PMiGAP), as described in Sehgal et al. [21] and Yadav et al. [22], were used in this study. These inbred lines represent the diverse pearl millet germplasm originating from 22 different countries including 40 accessions from ICRISAT, 15 accessions from India, 22 from Niger, 10 from Nigeria, 9 each from Namibia, Zimbabwe, Togo, and the remaining 52 from other pearl millet growing regions (Supplementary Table S1). The seeds of each of the 197 accessions were multiplied in the uniform field conditions at ICRISAT, Patancheru, India, following standard agronomic practices as described by Upadhyaya et al. [23], Ramya et al. [24], and Yadav et al. [22]. The seeds were collected after selfing under strict pollination conditions to obtain pure seeds of each line. The seeds were supplied to IBERS, Aberystwyth University, by ICRISAT via standard material transfer agreements.

### 2.2. Metabolomic Profiling

Metabolites were extracted from frozen milled samples of each accession of the PMiGAP and were performed in four replicates of each accession. The seed samples (~50 mg ± 1 mg) were placed into 2 mL microcentrifuge tubes, each containing a single 5 mm diameter stainless-steel ball (acetone cleaned). Samples were immediately flash frozen in liquid N_2_ and homogenized using a ball mill and put on ice to which 1 mL of extraction solution (chloroform/methanol/water, 1:2.5:1, v/v/v) was added followed by incubation at 4 °C for 15 min. The samples were further centrifuged at 14,000 rpm for 5 min at 4 °C and returned to the ice. The aqueous supernatant was transferred into new tubes for MS analysis. The extracted samples (100 µL) were transferred into the MS vials with inserts and analyzed for FIE-HRMS (flow infusion electrospray ionization high-resolution mass spectrometry). The four independent replicates were made for each sample. Metabolite fingerprinting was performed by FIE-HRMS using a Q Exactive Plus Hybrid Quadrupole Orbitrap Mass Analyser with an Acella ultra high-performance liquid chromatography (UHPLC) system (Thermo Fisher Scientific, Bremen, Germany). The sample was injected into the capillary column in a randomized order. The *m*/*z* (mass-ion) values were generated in both positive and negative ionization modes as described by Skalska et al. [20].

### 2.3. Statistical Analysis for Metabolites Identification

Individual *m*/*z* values were normalized with the Pareto scaling method and log_10_ transformed for each sample. Multivariate analysis was performed using MetaboAnalystR (http://www.metaboanalyst.ca (accessed on 29 September 2021). The significance of the cross validated *p*-values, based on the one-way analysis of variance (ANOVA) was set to *p* < 0.05. The multiple comparison and post hoc test using Fisher’s Least Significant Difference (Fisher’s LSD) were performed. The functional level and pathway enrichment assessment were performed using the Functional analysis module of MetaboAnalyst 5.0. Metabolite identification was based on the MS peaks to pathway algorithm (tolerance = 5 ppm, reference library: *Oryza sativa*).

### 2.4. Selection of Metabolites Contributing to Health Benefiting Traits

ANOVA using the Wald statistic in Genstat was used to calculate metabolite variance across 197 PMiGAP genotypes. The mean values were calculated for all 197 PMiGAP genotypes. The PMiGAP lines showed significant variation in the following: starch and sucrose metabolism; cutin, suberin and wax biosynthesis, antioxidants biosynthesis pathways (such as folate biosynthesis, inositol phosphate metabolism, flavonoid biosynthesis, thiamine metabolism, carotenoid biosynthesis, flavone and flavonol biosynthesis, zeatin biosynthesis, stilbenoid, diarylheptanoid, and gingerol biosynthesis); lipid biosynthesis (fatty acid degradation, fatty acid biosynthesis, biosynthesis of unsaturated fatty acids), vitamins (one carbon pool by folate, riboflavin metabolism, nicotinate and nicotinamide metabolism, linoleic acid metabolism, biotin metabolism, vitamin b6 metabolism) and nitrogen metabolism. Broad sense heritability (H^2^) was analyzed as described by Yadav et al. [22], and the best linear unbiased prediction (BLUP) value was estimated as defined in the user manual of HAPPI GWAS (Holistic Analysis with Pre- and Post-Integration GWAS) [25]. The BLUP values were used as the input data for metabolic genome-wide marker association studies.

### 2.5. Metabolic Genome-Wide Marker Association Studies

Metabolic genome-wide marker association studies (mGWAS) were performed using a mixed linear model (MLM) on an R platform implemented in GAPIT (Genomic Association and Prediction Integrated Tool) for each of the metabolites contributing to the identified nutritional traits [26]. MLM is a robust model for rectifying the fixed and random genetic effects and controlling the bias of population stratification while identifying associations between SNP polymorphisms and the traits [22]. Additionally, the model also reduces any confounding effect between testing markers using the kinship matrix (K) and Q matrix as random effects. SNP markers having a *p*-value < 0.001 were normally considered significant. For declaring marker-trait association to be highly significant, a higher threshold to −log10 ≤ 4.0 was set. Manhattan plots displayed statistically significant associated markers and quantile-quantile (Q-Q) plots were prepared to graphically visualize the distribution pattern for associated markers. The R squared values for markers (r^2^) were calculated and used to explain the proportion of phenotypic variation explained by each SNP locus.

### 2.6. Identification of Candidate Genes Affecting Metabolites Contributing to Health Benefiting Traits

The probable candidate gene search from the significant SNP-trait associations obtained from mGWAS was determined based on the extent of linkage disequilibrium (LD) surrounding the SNP. LD was calculated using D prime in a 10 kb window surrounding region of the significant SNP by Haploview software version 4.2 [27]. Regions of high LD (95% confidence bounds on D prime) (i.e., haploblocks) were identified and gene search has been performed using HAPPI GWAS (Holistic Analysis with Pre- and Post-Integration GWAS) [25]. Searches for candidate genes were performed using the gene annotated GFF file from the database (ftp://cegresources.icrisat.org accessed on 29 September 2021) according to the positions of the closest flanking significantly associated SNPs. SNPs that were significantly associated with the trait but were not falling within the regions of high LD, were not considered. The functions of corresponding genes were predicted using the Blast2 Go program [28]. Homology based identification of probable candidate genes associated with significant polymorphic SNPs were annotated using available databases (NCBI-nr, PIR, KEGG, and GO).

## 3. Results

### 3.1. Metabolite Fingerprinting of Pearl Millet Seeds

Metabolite fingerprinting was performed using FIE-HRMS (flow infusion electrospray high-resolution mass spectrometry) for 197 pearl millet accessions originating from different parts of the world. A total of 4189 mass features were observed of which 2227 mass features were in negative ionization mode and 1962 in positive ionization mode for the PGMiGAP lines.

**Figure 1:**
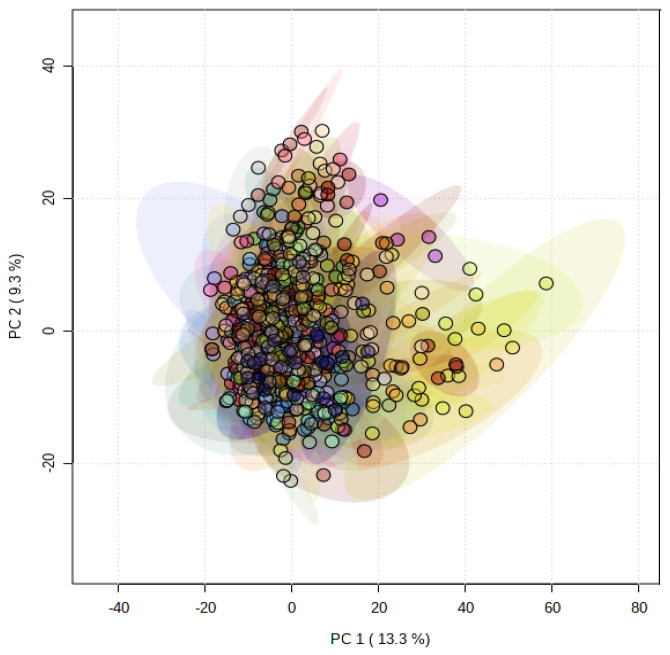
Principal component analysis (PCA) of metabolite profiles of 197 pearl millet accessions. Each ellipsis describes the variation within four replicates of a single accession.

Principal component analysis (PCA) showed that the metabolomes of the PGMiGAP lines exhibited no clear metabolomic sub-populations (Figure 1). The variation described by principal component (PC) 1 was 13.3% and overall, the first five coordinates accounted for a total of 40.7% variation. This indicated that there were relatively small differences in the global metabolomes in the PGMiGAP lines. This stated, ANOVA analysis along with multiple comparisons by false discovery rate (FDR) statistical approach, identified significant differences in individual metabolites among the 197 accessions studied (Supplementary Table S2). ANOVA analysis revealed that 1333 mass features (negative ionization mode) and 1162 mass features (positive ionization mode) significantly differed at *p* < 0.05 in PMiGAP lines. The pairwise comparisons were performed for such potential metabolites having significant differential quantities among the 197-pearl millet and shown in the heatmap and dendrograms (Supplementary Figure S1,S2). These suggested that the PGMiGAP lines could be broadly clustered into three clades.

### 3.2. Predictions of Metabolic Pathways for Each Metabolic Compound

**Figure 2:**
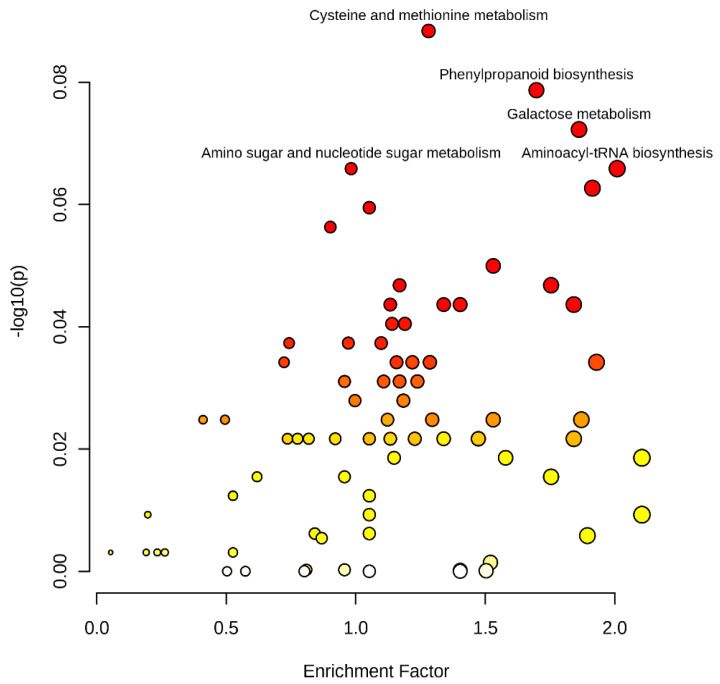
Metabolite pathway analyses showing metabolite enrichment and variation in pearl millet lines originated using the mummichog algorithm.

A total of 2295 (1333 mass feature with negative ionization mode and 1162 with positive ionization mode) significant mass features were subjected to metabolic pathway analysis for identifying their suggestive functional information. Metabolite pathway assessments using the mummichog algorithm (https://shuzhao-li.github.io/mummichog.org accessed on 29 September 2021) identified 1276 (694 negative ion mode and 582 positive ion mode) mass features showing significantly hits with KEGG pathway libraries at a cut-off *p*-value > 1 × 10^−5^. The most significant association are displayed in Figure 2. Important pathways identified were galactose metabolism (44 mass features), flavonoid biosynthesis (40) amino sugar and nucleotide sugar metabolism (37), phenylpropanoid biosynthesis (36), valine, leucine and isoleucine biosynthesis (30), pyrimidine metabolism (31), aminoacyl-trna biosynthesis (37), cysteine and methionine metabolism (35), alanine, aspartate and glutamate metabolism (24), starch and sucrose metabolism (24), purine metabolism (30), glycine, serine and threonine metabolism (36) and tryptophan metabolism (16). Less prominent, but biologically important, pathways included the TCA cycle, glycolysis/gluconeogenesis, citrate cycle (TCA cycle), pentose and glucuronate interconversions, valine, leucine and isoleucine degradation, alanine, aspartate, and glutamate metabolism.

### 3.3. Metabolites Contributing to Nutritional Health Benefiting Traits

Examination of the identified mass features (1276) allowed the identification of 341 health promoting metabolites (Supplementary Table S3). A total of 24 metabolites that were identified involved in starch and sucrose metabolism. Other pathway specific variation targeted included the following: 10 mass features associated with cutin, suberine and wax biosynthesis; 135 metabolites with antioxidants biosynthesis pathways (such as folate biosynthesis, inositol phosphate metabolism, flavonoid biosynthesis, thiamine metabolism, carotenoid biosynthesis, flavone and flavonol biosynthesis, zeatin biosynthesis, stilbenoid, diarylheptanoid, and gingerol biosynthesis); 68 with lipid biosynthesis (fatty acid degradation, fatty acid biosynthesis, biosynthesis of unsaturated fatty acids); 66 metabolites with vitamins (one carbon pool by folate, riboflavin metabolism, nicotinate and nicotinamide metabolism, linoleic acid metabolism, biotin metabolism, vitamin b6 metabolism) and 7 metabolites with nitrogen metabolism.

**Figure 3:**
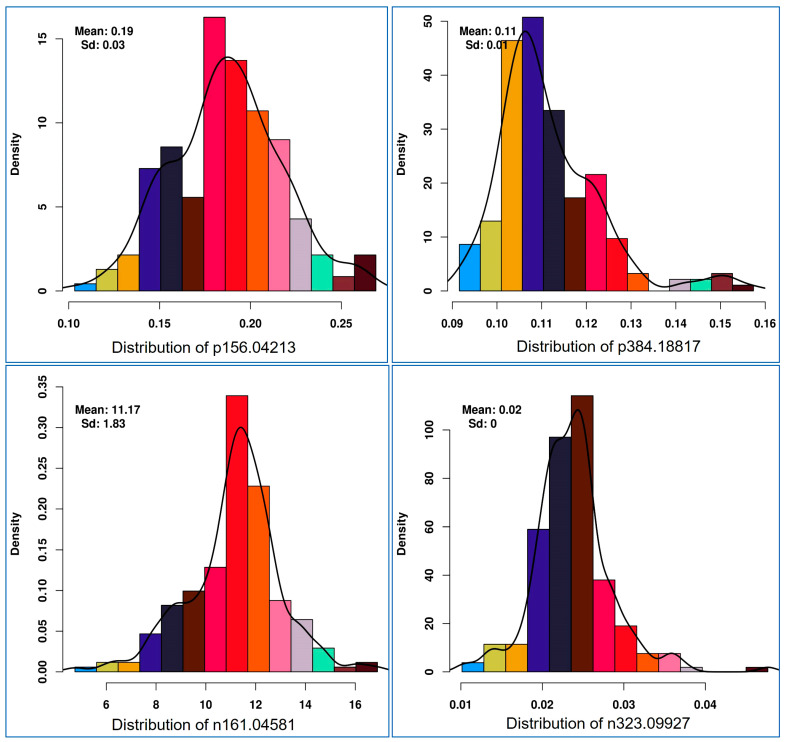
Histogram distribution pattern for selective mass features (p156.04213-in anthocyanin/lipid metabolism, p384.18817-vitamins metabolism, n161.04581-vitamins/starch metabolism, n323.09927-starch and sucrose metabolism) used for mGWAS (metabolic genome-wide association analysis).

Histogram plot of each health benefiting nutritional metabolite showed a normal distribution pattern for health promoting metabolites. In Figure 3, the patterns of four representative features (positive ion [p] 156.04213 *m*/*z*, p384.18817, negative ion [n] 161.04581 and [n] 323.09927) are shown. The heritability of each such nutritional metabolite was calculated, which ranged from 7.2% to 96.9%. Eight metabolic traits showed less than 50% heritability (Supplementary Table S3).

### 3.4. Metabolic Genome-Wide Marker Association Studies

mGWAS analyses were performed for 333 mass features using 76648 polymorphic SNPs, which were filtered following Yadav et al. [22] with minor allele frequencies (MAFs) < 0.05, or missing data > 20% from the peal millet 28 M SNP described in Varshney et al. [4]. A total of 6714 SNP showed significant association with 333 metabolites at the *p*-value of ≤ 0.001 (Supplementary Table S4). The metabolic variance (R2) explained by each locus ranged from 7.0 to 28.1%. Approximately, 30 metabolites (negative ion mode) were found to be linked with more than 10 loci during marker-trait association analysis (MTA) whereas 20 mass features (positive ionization mode) were influenced by more than 10 loci. One of the negative ion mode mass feature (n401.13098 likely to be flavanone 7-O-beta-D-glucoside) was genetically controlled by a maximum of 24 loci followed by n297.24417 (likely, oxooctadecanoic acid) with 20 loci. Similarly, the positive ion mode mass feature p496.34027; (likely, lysophosphatidylcholine (16:0)) was genetically controlled by a maximum of 16 loci followed by p86.06037 (likely, pyroglutamic acid) with 15 loci, p455.10834 (likely, apigenin 4’-O-glucoside) with 14 loci.

**Figure 4:**
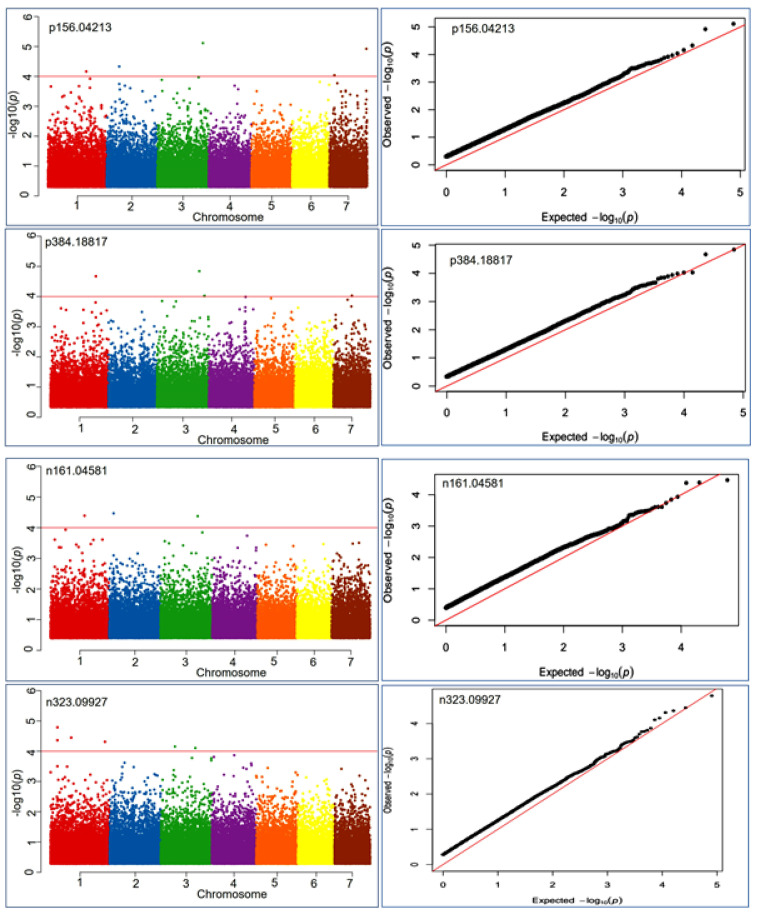
Manhattan and QQ plots exhibiting significantly associated SNP markers based on *p*-values measured by MLM model during metabolic genome-wide association studies (mGWAS) for four representative mass features p156.04213 involved in anthocyanin and lipid metabolism, p384.18817 involved in vitamins metabolism, n161.04581 involved in vitamins and starch metabolism, n323.09927 involved in starch and sucrose metabolism.

A total of 897 SNPs were found to be associated with health benefiting nutritional metabolite at the −log10 ≤ 4.0. Out of these, 616 SNPs were associated with negative ion mode and 333 SNPs with positive ion mode metabolic compounds (Supplementary Table S5). Thus, a total of 287 metabolite features had at least one associated locus at the significance level of -log *p*-value ≤ 4.0 in the mGWAS analysis. The markers were plotted against their chromosomal positions and the observed *p*-values (on a –log_10_ scale) to show the significantly associated markers. The Manhattan and Q-Q plot visualization indicated that significantly associated SNPs at the lowest *p*-value ranged from 9.9 × 10^−5^–2.1 × 10^−7^ (Figure 4). MLM-based association analysis demonstrated that the 2_210427657 locus exhibited highly associated MTA with the positive mode metabolite p611.16241 (most likely, rutin) at a *p*-value of 1.4 × 10^−7^. Similarly, the same locus (2_210427657) had a strong association with the negative mode metabolic compound n610.15161 (most likely, a hydrocinnamic acid compound) at a *p*-value of 2.1 × 10^−7^.

### 3.5. Suggesting Candidate Genes Affecting Metabolites Contributing to Health Benefiting Traits

The SNPs that are significantly associated with metabolites were annotated for probable candidate genes using the *Pennisetum glaucum* reference genome assembly (http://ceg.icrisat.org/ipmgsc/genome.htmL accessed on 29 September 2021). Putative genes surrounding the significant SNPs mapped within the regions of high LD blocks (r^2^ > 0.6) in 20 kb windows were considered as a probable candidate gene in mGWAS analysis. Genes contained or partially contained within the LD blocks were also considered as a trait associated candidate gene. If no genes overlap the haploblock, or the significant SNP does not fall within an LD block, the gene directly upstream and downstream of the significant SNP is given. In addition to genes that were the nearest neighbors to significant SNPs but not mapped in LD blocks, were not considered as candidate genes. Thus, seven hundred and thirty-eight candidate genes were identified in the surrounding regions of the significantly associated SNPs and high LD haploblock.

Out of 738 SNPs, 53 (0.07%) SNP’s adjoining genes were detected to be of similar functions to starch biosynthetic pathway-related genes. Among them, beta-amylase (Pgl_GLEAN_10022932, Pgl_GLEAN_10018323 and Pgl_GLEAN_10021788), alpha-amylase (Pgl_GLEAN_10005587 and Pgl_GLEAN_10031363) and starch synthase, catalytic domain (Pgl_GLEAN_10027180) were predicted to be involved in starch biosynthetic pathway-related genes (Supplementary Table S6). Mass features such as n217.02998 (likely to be a glucose adduct) showed significant association with SNP (1_224032459) at the *p*-value 7.7 ×10^−5^ and flanking gene (Pgl_GLEAN_10017489) was likely involved in starch and sucrose metabolism. Similarly, n99.04523 (likely, erythrose 4-phosphate) exhibited strong association with SNPs (2_17284984, 2_17284990, 2_17285011 and 2_17285041) at the *p*-value >2.4 ×10^−6^ and flanking gene (Pgl_GLEAN_10005402) had regulatory role in starch and sucrose metabolism. Likewise, mGWAS exhibited mass feature (n323.09927, likely to be sedoheptulose) showing a strong association with SNP (3_214922811) at the *p*-value 7.8^−5^ and functional annotation of a nearby gene (Pgl_GLEAN_10031396) encoding alpha-amylase/branching enzyme, having molecular active sites involved in the initiation and formation of starch granules and the conversion of amylose and amylopectin. Thus, the observations suggested that more than ten genes are having active roles in starch biosynthesis regulating the various chemical pathways to promote changes in sugar metabolism leading to the accumulation of amylose and starch. Several other candidate genes involved in starch and sucrose metabolism pathways were also detected during mGWAS, but these genes regulated distantly related metabolic pathways. For example, Pgl_GLEAN_10018323 encodes β-amylase involved in the chemical reactions and pathways resulting in the breakdown of a polysaccharide, a polymer of many (typically more than 10) monosaccharide residues was identified as a candidate gene associated with n183.00641 (likely to be 2-hydroxy-3-oxobutyl phosphate) having a critical role in plant vitamin metabolism. It was noteworthy that Pgl_GLEAN_10004380, which encodes lipase or esterase activities having an essential role in digestion, transport, and processing of dietary lipids, has been detected to be a probable candidate gene for mass feature n297.24417 (likely, oxooctadecanoic acid) involved in starch and lipid metabolism.

Approximately, 16 candidate genes having functional properties related to antioxidant compounds, exhibited a strong association with mass features having similarities with folate biosynthesis, inositol phosphate metabolism, flavonoid biosynthesis, thiamine metabolism, carotenoid biosynthesis, flavone and flavonol biosynthesis-related genes during metabolic pathway analysis. For example, SNP (6_106009267) flanking gene (Pgl_GLEAN_10010541) encoding carbamoyltransferase enzyme, predicted to be a candidate gene involved in antioxidants biosynthetic pathway, was found to have a significant association with mass feature n337.09402 (likely to be a chalone compound) involved in phenylpropanoid biosynthesis, stilbenoid, diarylheptanoid, and gingerol biosynthesis at the *p*-value ≥ 3.3^−5^. Similarly, GDSL/SGNH-like Acyl-Esterase (Pgl_GLEAN_10002072) encoding enzyme, having hydrolase activity and especially acting on ester bonds to promote the accumulation of carotenoids, showed significant association with mass feature p611.16241 at the *p*-value of ≥7.1 ×10^−5^. This suggests that these genes might be the key factors in these pathways through various chemical pathways to promote the accumulation of antioxidant-related flavonoid compounds.

A vitamin-K reductase (Pgl_GLEAN_10004357) gene on chromosome 7 was identified (at the *p*-value of ≥1.1 × 10^−5)^), which is known to be related to vitamin K biosynthesis. It was found adjacent to an SNP marker (7_149936526) showing significant association with p156.04213 and n281.03635 mass features involved in anthocyanin biosynthesis and lipid metabolism. Similarly, fifteen genes encoding UDP-glucuronosyl and UDP-glucosyl transferase (UGTs) were found to be involved in starch metabolism, anthocyanin, and flavonol. UGTs have been significantly associated with vitamin, antioxidant, starch, and lipid metabolism-related mass features.

L-ascorbate oxidase/ascorbase (Pgl_GLEAN_10037006) gene related to vitamins metabolism act as a cofactor for enzymes involved in regulating photosynthesis, hormone biosynthesis, and regenerating other antioxidants. mGWAS revealed that this ascorbate gene showed a strong association with n281.03635, which was annotated as a biotin metabolism-related compound. An important plant bioactive, isoflavone 2′-monooxygenase encoding gene (Pgl_GLEAN_10031980) having polyphenolic metabolites with antioxidant properties were detected to be significantly associated with p384.18817 related to zeatin biosynthesis metabolic pathway at the *p*-value of ≥9.3^−5^. Similarly, many candidate genes predicted to be associated with lipid metabolism-related genes were identified that encoded mitochondrial proteins or proteins linked to stress defense mechanisms, growth, and development. Other genes showing similarities with transcription factors, including MADS-box transcription factor, Nodulin-like, Myb domain transcript, and auxin-related transcriptome factors, were also identified (Supplementary Table S7).

## 4. Discussion

A number of research studies have shown that nutrition-rich diets made of millet grain offer protection against several non-communicable diseases such as cancer, cardiovascular ailments, diabetes, metabolic syndrome, and Parkinson’s disease [12,13]. Pearl millet grains possess antioxidants, polyphenols, vitamins, starch especially dietary starch in abundance. For the metabolome analysis, from among the several available analytical tools such as high-performance liquid chromatography (HPLC), liquid chromatography-mass spectrometry (LC-MS), gas chromatography-mass spectrometry (GC–MS), nuclear magnetic resonance (NMR), FIE-HRMS (flow infusion high-resolution mass spectrometry) available, the latter is known to offer greater advantages in capturing larger metabolomic information in a sample [29]. As a result, we employed FIE-HRMS to link health benefiting nutritional compounds to genetic elements in pearl millet. Thus, FIE-HRMS detected 333 mass features, which were crucial in determining health benefiting nutritional traits via biosynthesis of starch, antioxidants biosynthesis, lipid biosynthesis, and vitamin metabolism.

In this report, we employed mGWAS using MLM model to link 896 SNPs with health benefiting metabolites in a population of 197 pearl millet accessions. In the surrounding regions of these significantly associated SNPs, 738 probable candidate genes were identified that were linked to metabolic compounds. In doing so, we for the first-time report in pearl millet where metabolic trait-associated marker study was conducted using LD to identify candidate genes controlling health benefiting nutritional traits. In fact, the relationships between metabolic compound accumulation and their regulation at the genetic level has frequently been applied to study human diseases, but have only rarely been applied to plants [30]. These limited studies pertain to both the model plants as well as to some economically relevant crop species [31,32,33,34,35], however, the identification of the genetic bases of metabolites content using metabolic association analysis have only been reported in maize [30,36].

The starch biosynthetic pathway genes we report in this study would help to derive pearl millet varieties that are rich not only in dietary starch, but also having the characteristics such as low GI and high fiber content (β-glucans). Availability of such varieties will have hypoglycemic effects when consumed, and hence will be beneficial in preventing type-2 diabetes. Further, in this study, mGWAS was employed that identified SNP markers associated with starch biosynthesis-related mass features in pearl millet. A total of 53 candidate genes were identified in the flanking regions of associated SNP markers that were significantly associated with starch biosynthesis-related mass features. Among these candidate genes, alpha-amylase/branching enzyme and beta-amylase were found across all selected SNP data sets linked to the starch related metabolic compounds. Our study corroborates with the findings of Zhang et al. [37], who also previously reported that starch branching enzymes IIa (BEIIa) was closely associated with starch traits in Indica rice. In another study, Parween et al. [38] also suggested alpha-amylase/branching enzyme having a key regulatory gene contributing to enhance resistant starch in rice. We also report another important gene having a role in digestion, transport, processing of dietary starch and lipids (encoded by a lipase gene, Pgl_GLEAN_10004380) linking with the accumulation of metabolite (n297.24417 oxooctadecanoic acid) involved in starch and lipid metabolism. This corroborates with Zhang et al. [37], who also reported that a lipase gene (LOC_Os09 g09360) was associated with slowly digestible starch through their GWAS study in rice. We have also identified several other starch synthase-related genes, especially the UTP-glucose-1-phosphate uridylyltransferase (UGT), having a role in starch-related compound accumulation.

Apart from dietary starch, plant bioactive metabolites and associated isoflavones are also equally beneficial for human health. Specifically, they reduce the risk factors for cardiovascular diseases including lowering liver or blood triglyceride, total and LDL cholesterol levels. In this study, using mGWAS we have identified SNPs influencing the antioxidants related bioactive compounds and 16 probable candidate genes including isoflavone 2′-monooxygenase encoding gene, carbamoyltransferase, GDSL/SGNH-like acyl-Esterase, and UGTs, which have similarities with the antioxidant biosynthetic pathway-related genes. These genes have also previously been reported to be associated with antioxidant metabolism-related pathways using an association analysis in pearl millet [22]. In the present study, we also elucidated more than ten pearl millet genes involved in biosynthesis of plant vitamins such as biotin, ascorbic acid, zeatin, riboflavin and thiamine related metabolic compounds. These corroborate findings by Luo et al. [39] in maize who identified SNP markers and ZmVTE4 genes associated with phenotypic variation of vitamin E production.

## 5. Conclusions

Traditional breeding using marker technology is mainly based on the selection of agronomic traits using linked DNA markers, but the availability of metabolites-based biomarkers offers an additional toolbox to the plant breeders. Synthesizing a new variety for crop plants such as of rice, wheat or millets generally requires 5–7 years through traditional breeding schemes and any approach that can hasten such process is desirable. Traditional breeding schemes also suffer from a lack of precision as selection based on phenotype is not only time and labor-consuming, but poor, especially when traits under selection are complex. We demonstrate that by combining metabolite-based phenotyping in conjunction with genotypic data, molecular markers associated with health benefiting nutritional traits can be identified. Identification of candidate genes and metabolites regulating such health benefiting traits as reported in this study will go a long way in assisting breeding of useful health benefitting traits in crops plants.

## Data Availability

The data presented in this study are available as Supplementary Files.

## Supplementary Materials

The following are available online at https://www.mdpi.com/article/10.3390/cells10113076/s1, Table S1: Details of the different 197 accessions which is part of pearl millet Inbred Germplasm Association Panel (PMiGAP) lines originated from different parts of the world. Table S2: Significant mass features by ANOVA analysis with a *p*-value threshold of 0.05 correcting for false discovery rates (FDR) in pearl millet germplasm association panel (PMiGAP) of 197 genotypes. Table S3: Heritability and ANOVA results for 341 mass features (209 positive ion mode and 132 positive ion mode) in 197 pearl millet lines during metabolic genome-wide association studies. Table S4: Metabolite profiling for 341 mass features (209 positive ion mode and 132 positive ion mode) considered as health benefiting nutritional traits in 197 PMiGAP pearl millet germplasm. Table S5: Significantly associated SNP markers with health benefiting nutritional traits using 76 K SNPs in pearl millet analyzed through MLM based statistical model on a collection of 197 individuals of pearl millet. Table S6: List of health benefiting metabolic pathway-related candidate gene resided around significantly associated markers in pearl millet. Table S7: Probable candidate genes resided around significantly associated markers with metabolite in pearl millet. Figure S1: The heatmaps exhibit differential metabolites in pairwise comparison among the 197 pearl millet accessions. Figure S2: Dendrogram analysis showing distinct metabolites clustering among the 197 pearl millet accessions.
