# Supplementary figures and images for: Metabolite Diversity and Metabolic Genome-Wide Marker Association Studies (Mgwas) for Health Benefiting Nutritional Traits in Pearl Millet Grains
Source: Cells. 2021 Nov 8;10(11):3076. doi: 10.3390/cells10113076 (PMC8621611; doi:10.3390/cells10113076)

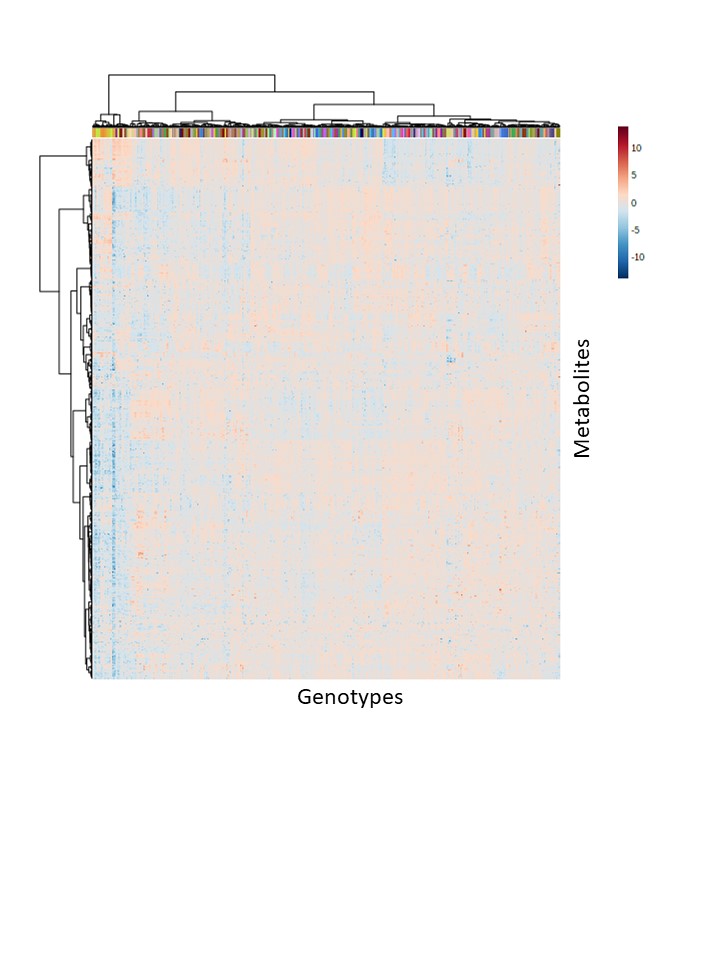

Supplement: Supplementary file 1 [file cells-10-03076-s001.zip › Suppl_Figure S1.jpg]
